# Circular reasoning concerning Red flags for predicting rituximab response in OCD

**DOI:** 10.1038/s41380-024-02885-y

**Published:** 2025-01-03

**Authors:** Susanne Bejerot, Albert Max Hietala, Annika Söderbergh, Mats B. Humble

**Affiliations:** 1 Faculty of Medicine and Health, University Health Care Research Centre, Örebro University, Örebro, Sweden; 2 Faculty of Medicine and Health, School of Medical Sciences, Örebro University, Örebro, Sweden; 3 Department of Neurology, Karolinska University Hospital, Stockholm, Sweden; 4 Department of Rheumatology, Örebro University Hospital, Örebro, Sweden

**Keywords:** Predictive markers, Psychiatric disorders

Gallwitz et al. recently presented three cases of chronic obsessive-compulsive disorder (OCD) in adults, demonstrating improvement following repeated rituximab treatments [1]. However, the study does not provide the total number of evaluated or excluded patients, which limits the broader applicability of their findings. The authors suggest that a subset of OCD cases with immunological etiology, identified through at least three specific “red flags”—which include clinical symptoms and biological markers from cerebrospinal fluid (CSF) or blood—might benefit from repeated administration of rituximab, a B-lymphocyte depleting agent.

We acknowledge the innovation behind the authors’ immune marker analyses and agree that the patients they described were indeed responsive to treatment. Additionally, we concur that multiple rounds of rituximab are likely required to determine the overall efficacy of this intervention. Nonetheless, the authors’ reasoning contains significant logical shortcomings. They argue that clinical and biological markers can predict treatment outcomes, yet their sample only included individuals identified as having a “probable autoimmune subtype” based on the presence of these markers. By excluding patients without these markers, it remains unclear whether individuals without an autoimmune subtype of OCD might also respond to rituximab. To address this gap, a more informative initial approach would involve conducting pilot studies with rituximab on unselected patients, as we undertook between 2019 and 2021. Our studies included patients with treatment-resistant OCD (Rits-PO study, *n* = 10) or schizophrenia (Rits-PS study, *n* = 9), all of whom underwent extensive clinical interviews. These included the PsychoNeuroInflammatory related Signs and Symptoms Inventory (PNISSI), designed to identify clinical indicators of inflammatory subtypes in psychiatric disorders, as described elsewhere [2]. Blood samples and, in a subgroup of patients, CSF were collected for cytokine and other inflammatory marker analysis. Additionally, MRI brain scans were conducted. Due to ethical regulations, participants were administered only one single intravenous dose of rituximab (1000 mg) as an adjunct to their regular medication. The primary endpoint was set at five months, though patients were monitored for one year. Our results indicated that 2 out of 10 OCD patients showed significant improvement according to the Clinical Global Impression - Improvement (CGI-I), compared to 4 out of 8 schizophrenia patients [3]. Moreover, we observed a faster therapeutic response in schizophrenia patients compared to the OCD patients, along with changes in brain connectivity and morphology in the responders [4].

One of our OCD participants had been treated by Dr. Cunningham (the senior author of the Gallwitz et al. study) prior to her inclusion in our trial. This patient had been excluded from their study for not meeting the criteria for autoimmune OCD, as she lacked the requisite red flags. However, as we did not impose such thresholds, she was included in our Rits-PO study in 2019. This patient had developed acute-onset OCD at the age of 14, following a throat infection. By her late teens, her symptoms had severely worsened, with compulsions lasting more than three hours daily. She could no longer use a conventional bathroom, instead relieving herself outdoors, and avoided public transportation due to her obsessions. Dependent on her parents, she was also severely depressed and disengaged. Cognitive behavioral therapy (CBT) and pharmacological treatments (sertraline, clomipramine, and aripiprazole) had been ineffective. At the time of her inclusion in our study, she was only receiving low-dose clomipramine (25 mg) and antibiotics (amoxicillin). At the five-month endpoint, she showed minimal improvement and was categorized as a non-responder, though she was less fatigued, more active, and generally happier than at baseline. These improvements were difficult to quantify; her Yale-Brown Obsessive Compulsive Scale (Y-BOCS) score remained unchanged at 25 points, likely due to ceiling effects, and her Personal and Social Performance (PSP) score only increased marginally, from 40 to 45. At the end of the year, the patient experienced a deterioration in symptoms. However, due to her minimal prior response to rituximab, she was accepted for repeated treatments at another clinic (500 mg every six months). Gradual improvements were observed, and her OCD symptoms have significantly diminished after five rounds of treatment since 2022. Currently, she remains off medication (except for yearly rituximab) and is successfully participating in CBT, with plans to reenter the workforce. She is now a happy, independent young woman.

Another patient, not treated by us, had a typical gradual childhood onset of mild OCD and a strong familial predisposition, with two siblings also affected. She had no comorbid somatic conditions until her early twenties when she developed neuritis of the axillary nerve of unknown etiology. Approximately five years later, her OCD symptoms worsened, and she was diagnosed with multiple sclerosis (MS) in her mid-thirties. Following two doses of rituximab for MS, her OCD symptoms subsided entirely alongside improvements in her MS symptoms within a year. It is possible that the neuritis was a red flag for immune involvement, though she would likely not have been classified as having autoimmune OCD.

A third patient, our index case for investigating rituximab in psychiatric disorders, had suffered from severe, complex OCD since age 17 [5]. Despite extensive neurological evaluations, no signs of autoimmune encephalitis were detected until eight years after onset, when she was diagnosed with neuromyelitis optica spectrum disorder (NMOSD). As part of her treatment for NMOSD, she received rituximab, which led to the complete remission of her OCD and other comorbid psychiatric symptoms. She has now been successfully treated with rituximab every six months for ten years.

Among the schizophrenia patients in our Rits-PS study, one has continued rituximab treatment for five years. This patient demonstrated dramatic improvement at the five-month endpoint and, from being a frequent psychiatric inpatient, she is now capable of managing her life independently. Her treatment resistance and abrupt symptom onset would have been considered two red flags.

Currently, we are conducting a large, multicenter, double-blind, placebo-controlled trial (RCT-Rits) involving a single dose of rituximab (1000 mg) as an adjunct to antipsychotics in unselected schizophrenia spectrum disorder patients across Sweden [6]. Patients in Örebro County are offered the option of an open rituximab trial after the six-month endpoint.

To determine whether extensive investigations including CSF sampling are predictive for response, requires a different design (RCT) and a much larger study population than the three patients presented by Gallwitz et al. Excluding patients on the basis of the absence of biomarkers at an early stage of research may be premature and potentially misleading and has been previously criticized in another context [7].

Ultimately, circular reasoning serves to reinforce pre-existing conclusions, but does little to advance our understanding or improve patient outcomes.

## Data Availability

Data is available on request from the corresponding author (S Bejerot). All three cases have given approval of their respective case presentations.
